# Cationic Moieties in Polystyrene Gels Swollen with d-Limonene Improved Transdermal Delivery System

**DOI:** 10.3390/polym10111200

**Published:** 2018-10-27

**Authors:** Preeyarad Charoensumran, Hiroharu Ajiro

**Affiliations:** 1 Graduate School of Materials Science, Nara Institute of Science and Technology, 8916-5 Takayama-cho, Ikoma, Nara 630-0192, Japan; preeyarad.charoensumran.pz9@ms.naist.jp; 2 Institute for Research Inititatives, Nara Institute of Science and Technology, 8916-5 Takayama-cho, Ikoma, Nara 630-0192, Japan

**Keywords:** organogel, d-limonene, chemical enhancer, transdermal drug delivery

## Abstract

d-limonene, a terpene and natural compound, has been found to be an excellent penetration enhancer for transdermal drug delivery (TDD). It hence has been incorporated within various transdermal formulations. Herein, we report the application of polystyrene gel swollen with d-limonene and its derivatives for TDD. Poly(styrene-*co*-divinylbenzene) (PS gel), poly(styrene-*co*-divinylbenzene-*co*-4-vinylpyridine) (PS-4VP) gel and poly(styrene-*co*-divinylbenzene-*co*-(vinylbenzyl) trimethylammonium chloride) (PS-VBAC gel) were employed as chemical gels to improve the stability of the TDD substrates. The drug permeation properties from the PS gels swollen in limonene were examined, regarding the effect of its network density as well as their rheological properties. The lowest density of the network showed the highest steady flux of the permeation at 43.7 ± 0.3 μg/cm^2^. FT-IR spectra were confirmed for PS-4VP and PS-VBAC, bearing cationic moieties and they could control the release of ibuprofen by the electrostatic interaction at the interface of organogel and skin. The steady state flux of skin permeation got low values from 55.2 ± 0.8 to 11.6 ± 2.0 μg/cm^2^, when the cationic moieties were increased. Moreover, the chemical network of PS gel swollen in limonene showed high mechanical stability illustrated by elastic modulus (G’) of about 98 kPa for 10% cross-linked PS gel. The developed PS gels swollen in limonene show highly promising results, suggesting their possible application in TDD.

## 1. Introduction

Transdermal drug delivery (TDD), the transportation of drugs across the skin, has been an attractive research area because of its obvious advantages over other routes of delivery [1,2]. Transdermal delivery systems provide convenient, pain-free and self-administrated use for the patient. It avoids the gastrointestinal side effects, usually entailed by many oral preparations. TDD also avoids fluctuations in plasma drug concentration, which helps minimizing adverse effects and therapeutic failure.

The main challenge in TDD, however, is to overcome the inherent barrier of the skin. It has been reported that the rate limiting step in transdermal delivery is the ∼30 μm thick stratum corneum which acts as a protective barrier against exogenous molecules including drugs [3]. For this reason, a variety of molecules [4] and materials [5] have been investigated as candidates to enable or facilitate skin permeation. 

Chemical penetration enhancers have been widely used to increase the skin permeability of many therapeutic molecules and anesthetics [6] by interacting with the stratum corneum (SC) lipid or keratin [7], or by increasing the solubility of drug into SC lipid [8,9]. Extensive research during the past two decades has led to the formulation of several different classes of penetration enhancer such as terpene compound. Many reports have already provided substantial evidence that terpene [10,11] are capable of enhancing percutaneous transportation, especially d-limonene. Zhen Yang and co-workers [12] has been reported the d-limonene was the most effective permeation enhancer (PE) to enhance skin permeation of bufalin among other terpene compound and different synthetic PEs.

D-limonene, is a neutral-derived terpene compound which is well known as permeation enhancer in transdermal delivery system [13,14]. It is listed in the Code of Federal Regulations as generally recognized as a safe (GRAS) [15]. Moreover, it has low toxicity, so it is appropriate to boost up the transdermal delivery system 

Regarding the material side, organogels are promising candidates for drug delivery system including dermal and transdermal application [16,17], because of their intrinsic properties. They are lipophilic, non-irritating easy-to-use and moisture insensitive. In an earlier work, Chan and co-workers reported the preparation of limonene PG1/propylene glycol organogels as a physical gel [18]. However, it has a limitation on increasing the amount of limonene because it affects the stability of organogels. Yang and co-workers reported transdermal delivery of ibuprofen using microemulsion as a vehicle [19]. Microemulsions are spreadable materials requiring a specific ratio of the oil-surfactant-water system. In order to open the door to facile preparation, we suggest that the convenient use and stability against various conditions, organogels as a chemical gel swollen with limonene [20] are an auspicious approach to solve the limitation of TDD in both of applications and permeability.

Actually, it is known that the d-limonene is the excellent solvent for polystyrene (PS) [21] which is well known aromatic polymer. In addition, it can use as eco-friendly solvent for dissolving wasted expanded PS [22]. According to this advantage, the network structure consists of cross-linked PS gel is possible to swollen in d-limonene. Since PS has hydrophobic and biocompatible properties [23], it can be widely used in a variety of applications such as adsorbent materials in pollutant recovery [24,25], drug storage to control release for lipophilic compounds [26] and transdermal delivery systems [27,28].

In this work, the cross-linked polystyrene swollen in d-limonene as reservoir-type transdermal system was studied. We developed transdermal materials by using d-limonene as solvent for chemical organogel because of its chemical enhancer property and We investigated the permeation behavior of ibuprofen via the limonene gel through skin in this study in order to clarify the controlled release by electrostatic interaction of cationic moiety on the surface, although the limonene gel as drug reservoir will be study with adhesive membrane to abate effect of limonene contact to skin in the future. Herein, effect of network density of PS gels was studied by rheological measurements, as well as its influence on permeability and controlled release behaviors. The cationic moieties, 4-vinylpyridine (4VP) and vinylbenzyl trimethylammonium chloride (VBAC), were selected as interaction units with drug molecules for the prolong release. The interaction between drug and cationic moieties were observed by FT-IR. The relationship between elastic moduli and permeability were also discussed.

## 2. Materials and Methods

### 2.1. Materials 

St (99.0%), azobisisobutyronitrile (AIBN) (98.0%) and PBS buffer solution (×10) were purchased from Wako Pure Chemical Industry Ltd., Osaka, Japan. Super dehydrated toluene (99.5%), divinylbenzene (DVB) (50.0%), 4-vinylpyridine (4VP) (95.0%), Sodium 4-styrenesulfonate (93.0%), Acetonitrile (99.0%) and isobutylphenyl propionic acid (Ibuprofen,> 98.0%) were all purchased from Tokyo Chemical Industry Co., Ltd., Tokyo, Japan (TCI). d-limonene (90.0%), tetrahydrofuran (98.0%), dimethyl sulfoxide (99.0%) and bromocresol purple were purchased from Nacalai Tesque Inc. Kyoto Japan. Rhodamine B was purchased from Sigma Aldrich Co., St.Louis, MO, USA. Vinylbenzyl trimethylammonium chloride (VBAC) was purchased from Santa Cruz Biotechnology, Kyoto, Japan. All chemicals were used as supplied without further purification, except where noted otherwise.

### 2.2. Preparation of Organogels

The transdermal patch gel was prepared by cross-linked polymers swollen with d-limonene as the solvent. All monomers were purified by distillation to remove inhibitor before the polymerization. Firstly, as model drug, Ibuprofen solution in d-limonene (33.3 mg/mL) was prepared. Then, St, DVB as cross-linker and styrene derivative (SD) that is 4VP or VBAC, as cationic moieties were added. After that, 2.5 mol % of AIBN was added and the solution was sonicated for 5 minutes. In the following step, mixture was deoxygenated by nitrogen bubbling and heated at 60 °C for 24 h. The polymer network was radical polymerized, providing PS_n_-DVB_m_-SD_p_ while n, m and p refer to the feeding ratio of St, DVB and SD, respectively. The gels in this study are different from those reported in previous study [20], because PS gels were prepared in d-limonene while previously reported PS gels were prepared in toluene [20]. The gels were removed from the container and cut into 13 mm-diameter, 2 mm-thick discs (*V* = 0.256 cm³) for permeation test with a Franz diffusion cell. The drug concentration per disc was calculated from the total prepared gel containing 31 mg/cm³ of Ibuprofen, thus, each disc was found to contain 8.23 mg.

### 2.3. Swelling Property 

The PS gels swollen in limonene were freeze-dried after limonene had been washed out by benzene. Swelling properties of the synthesized gels were determined after 24 h. re-swelling in limonene by the following Equation (1):

(1) $$\mathrm{Swelling}\;\mathrm{ratio}\;(Q)=\frac{\left(W_{s}-W_{d}\right)}{W_{d}}\;$$

where *W*_s_ stands for the weight of the swollen gel in limonene and *W*_d_ for the weight of the dried gel after freeze-dried.

### 2.4. Rheological Study

The rheological properties of the PS gels swollen in limonene [20] were measured using a Rheometer (KNS2100, Kinexus; Malvern, UK). The organogels were cut in discs with 20 mm diameter and ~4 mm thickness and placed between two plates while the lower plate is fixed and the upper circle plate (20 mm diameter) is connect with the measuring system. The elastic modulus (G’) and viscous modulus (G’’) of the organogels swollen with limonene were measured at controlled frequency from 0.1 to 10 Hz at 25 °C in triplicate.

### 2.5. Preparation of Rat Skin

The rat skin was received from System Neurobiology and Medicine Laboratory, NAIST, Japan. All relevant aspect of experiment was approved by the Institutional Animal Care and Use Committee of Nara Institute of Science and Technology (reference No. 1802, approved on 13 March 2018). The abdominal skins of female rat (adult pregnant Wistar rat, weighing 250–300 g) were excised after sacrifice by cervical dislocation of rat. Adhering fat and other visceral debris were carefully removed. The processed skin was cut into pieces of appropriate size and used freshly without storing.

### 2.6. In Vitro Skin Permeation Study

In vitro skin permeation study [20] was carried out by using Franz-cell diffusion with receptor volume of 10 mL and an exposed area of 1.33 cm^2^. The 10 mL pH 7.4 phosphate buffer saline (PBS) was considered as receptor medium for the maintenance of physiological environment. The prepared skin sample was then mounted between acceptor and donor compartment of the cell and clamped with its dermal side in contact with the receptor medium. The prepared 13 mm diameter PS gel swollen in limonene was placed into the donor chamber. The diffusion cells were keep in 37 °C incubator. At designed time interval, 1 mL receptor medium was withdrawn from the receptor chamber and immediately replaced with the same amount of fresh PBS solution. 

### 2.7. HPLC Measurements for Drug Assay

Ibuprofen (isobutylphenyl propionic acid) concentration was determined with a reverse phase high performance liquid chromatography (HPLC) from Shimadzu; Kyoto, Japan. HPLC system using Cosmosil Packed Column 5C_18_-MSII column (4.6 mm × 150 mm, 5 μm). The detection condition was 60% PBS in acetonitrile as mobile phase with UV detector at 223 nm, 40 °C The flow rate was set as 0.6 mL/min and 100 μL of sample was loaded. Under these conditions, the resolution time of ibuprofen was 4.97–5.02 min. A calibration curve was constructed by using ibuprofen standard solution in PBS solution from 6.5–46 μg/mL (*R*^2^ = 1) shown in Figure S1.

### 2.8. Calculation of Permeation Parameter

The cumulative amount of drug (Q) permeating through the skin from the donor chamber at constant concentration (*C*_0_) to the receptor phase at the sink condition can be described by Fick’s 2^nd^ law of diffusion, Equation (2) [29] where *A* is the surface area, *L* is the thickness of the skin and *K* is the diffusion coefficient of the skin. Permeation parameters are interpreted from a cumulative drug per unit skin area Q/A versus time *t* plot. The steady-state flux (*J*_ss_) and lag time *t*_L_ were obtained from slope and x-interception value of the linear portion. The flux *J*_ss_ over drug concentration *C*_0_ in the donor solution gives permeability coefficient *KD*/*L* [30]. Diffusion parameter d/*L*^2^ reflects the mobility of the drug solute in the skin [31].

(2) $$Q={AKLC}_{0}\left[\frac{D}{L^{2}}t-\frac{1}{6}-\frac{2}{\pi^{2}}\sum_{n=1}^{\alpha }\frac{{\left(-1\right)}^{n}}{n^{2}}e^{-\left(\frac{D}{L^{2}}\right)n^{2}\pi^{2}t}\right]\;$$

(3) $$\frac{KD}{L}=\frac{J_{\mathrm{ss}}}{C_{0}}\;$$

(4) $$\frac{D}{L^{2}}=\frac{1}{t_{L}6}\;$$

## 3. Results and Discussion

### 3.1. Synthesis and Characterization of Limonene Organogels

**Figure 1:**
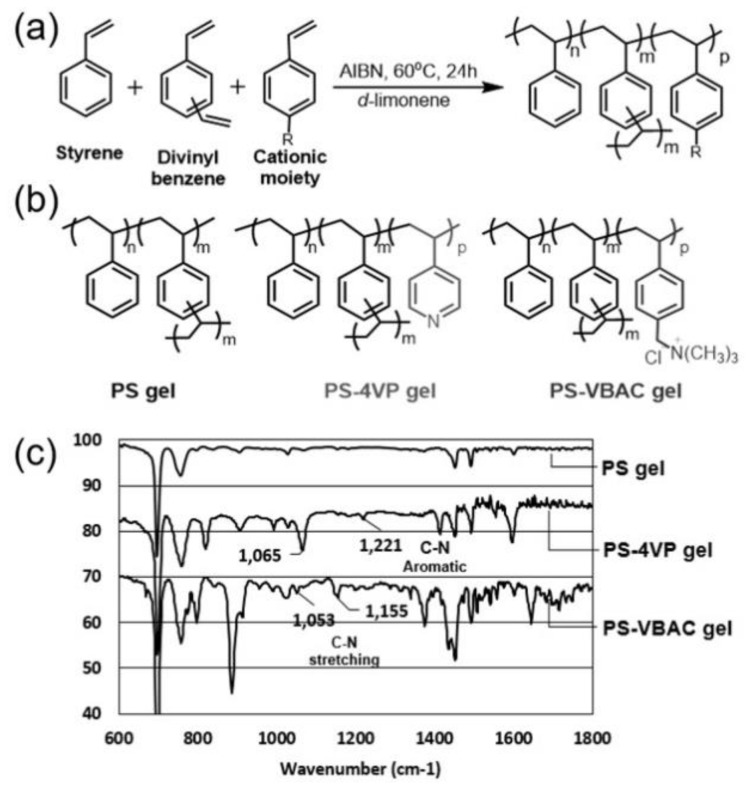
(**a**) Preparation of cross-linked PS gel in d-limonene. (**b**) Chemical structure of cross-linked PS gels. (**c**) FT-IR spectra of dry PS gel, PS-4VP gel and PS-VBAC.

The limonene gels, poly(styrene-*co*-divinylbenzene) (PS gel), poly(styrene-*co*-divinylbenzene-*co*-4-vinylpyridine) (PS-4VP gel) and poly(styrene-*co*-divinylbenzene-*co*-(vinylbenzyl) trimethylammonium chloride) (PS-VBAC gel) were prepared in d-limonene via radical polymerization (Figure 1a,b). The prepared gels in this study were listed in Table 1. The PS gels were prepared by varying the cross-linker ratio between 5 and 10 mol % and the concentration of d-limonene between 4 and 8 M resulting in gel A-D (Table 1, entries 1–4). These gels were used to study the effect of the network density. As a comparison, the PS gel was prepared in toluene as solvent for gel E (Table 1, entry 5). Moreover, the 4VP which acts as cationic moiety was introduced at 2–5 mol % being PS-4VP gel in gel F-J (Table 1, entries 6–10). As the other cationic moiety, the VBAC was introduced 0.5 and 1 mol % being PS-VBAC gel in gel K and L (Table 1, entries 11 and 12). Their elastic modulus (G’) were also listed in Table 1. The existence of cationic moieties was verified by FT-IR spectra of dry gels in Figure 1c. The PS-4VP gel, bearing pyridine group, was shown characteristic of C–N aromatic stretching at 1221 cm^−1^ and C–N stretching at 1065 cm^−1^. The PS-VBAC gel contained trimethyl ammonium group illustrated C–N stretching at 1053 and 1155 cm^−1^.

The 4VP and VBAC served as interaction unit in the copolymer for the controlled release by interacting with the drug. The positively charged VBAC would be expected to show stronger interaction with ibuprofen than 4VP (Figure S2). According to the interaction between drug and polymer chain, it could release ibuprofen through the skin in a controlled manner.

### 3.2. Swelling Behavior and Rheological Property of PS Gels Swollen in Limonene

**Figure 2:**
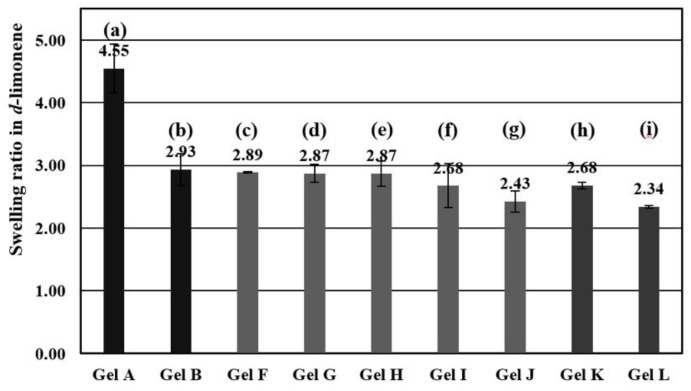
Swelling ratios of gel A (**a**), Gel B (**b**), Gel F (**c**), gel G (**d**), gel H (**e**), gel I (**f**), gel J (**g**), gel K (**h**) and gel L (**i**) (*n* = 3, error bars present standard deviation).

All gels were prepared directly by polymerization in d-limonene as solvent due to the good solubility of St in limonene [19,20]. Swelling ratios of various compositions of gels were determined as shown in Figure 2. The cross-linker amount of PS gel was compared at DVB contents of 5 and 10 mol % to evaluate the effect of the cross-linking degree on the density of the network structure and swelling properties. The swelling degree was decreasing as expected upon increasing the amount of cross-linker from 4.5 and 2.9 for 5 and 10 mol %, respectively (Figure 2a,b). These results confirmed the formation of a denser network structure when the cross-linking degree of PS gel was increased. Furthermore, the swelling ratio of PS-4VP gel (gel F-J in Figure 2c–g) and PS-VBAC gel (gel K and L in Figure 2h,i) with 5 mol % cross-linker were determined. The swelling ratios of PS-4VP gels were not significantly affected by enhancing of 4VP ratio in a range of 2–3 mol % (Table 1, entries 6–8), illustrating a swelling ratio of about 2.9 (Figure 2c–e). However, the swelling ratio of 4 and 5 mol % (Table 1, entries 9 and 10) of 4VP (gel I and J) were slightly decreased to2.68 and 2.43, respectively (Figure 2f,g). The shrinkage of gel I and J was caused by the low solubility of 4VP in d-limonene. In the same way, the VBAC was introduced at only 0.5 and 1 mol % (Table 1, entries 11 and 12). Their swelling ratio was calculated as 2.68 and 2.34, respectively (Figure 2h,i). As a result, the swelling ratio of PS gel was about 1.5 time higher for the cationic gels as PS-4VP gel and PS-VBAC gel which were referred to limitation of solubility in limonene.

**Figure 3:**
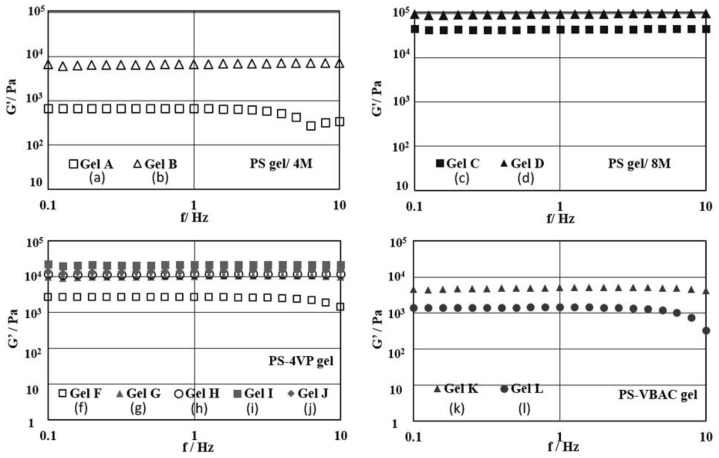
Elastic modulus (G’) from rheological measurement at frequency 0.1–10 Hz of 5 mol % cross-linker PS gel; Gel A (**a**) and Gel B (**b**), 10 mol % cross-linker PS gel; Gel C (**c**) and Gel D (**d**), PS-4VP gel; Gel F (**f**), Gel G (**g**), Gel H (**h**), Gel I (**i**) and Gel J (**j**) and PS-VBAC; Gel K (**k**) and Gel L (**l**) in d-limonene, (*n* = 3).

Previously, the rheological analyses on PS gels has been reported [20]. In this study, we further investigated the rheological properties with the detailed crosslinking and comonomer’s ratios regarding the PS gels obtained in d-limonene (Figure 3). The elastic modulus (G’) of gel B (Figure 3b) showed higher than that of gel A (Figure 3a), based on the higher crosslinking degree under 4 M condition. The same tendency was recognized between gel D (Figure 3d) and gel C (Figure 3c) under 8 M condition. Gel D illustrated the most rigid network structure providing an elastic modulus of 98 kPa at a frequency of 1 Hz (Figure 3d). This is about 2 times higher than gel C of 44 kPa (Figure 3c) and about 6 times higher than gel B of 6.7 kPa (Figure 3b). On the other hand, the concentration of the gel effects the strength of the network structure more than the cross-linking control as indicated by the elastic modulus.

It is shown that elastic moduli of the cationic gels, PS-4VP gel as gel F-J from 2–5 mol %-4VP (Figure 3f–j) and PS-VBAC gel as gel K and M between 0.5–1 mol %-VBAC (Figure 3k,l). The addition of 2% 4VP improved the elastic modulus from 680 Pa to 2600 Pa, when gel A and gel F were compared (Figure 3a,f). While the amount of 4VP in gel increased from 2 to 4 mol % (Table 1, entries 6–9), the G’ value gradually increased from 2600 Pa to 21 kPa (Figure 3f–i). However, the increasing ratio of 4-vinylpyridine until 5 mol % (Table 1, entry 10), the elastic modulus dropped from gel I which is 21 kPa to 14 kPa (Figure 3j). These results suggest that there is an optimized value for the strongest composition due to the complicated factors, such as electrostatic repulsion and the composition of monomers. Correspondingly, introducing a higher ratio of VBAC resulted in a lower elastic modulus in PS-VBAC gel (Table 1, entries 11 and 12), resulting from stronger repulsive forces of positively charged ammonium making the gel softer (Figure 3k,l).

After the evaluation of mechanical strength, the influence of the cationic moieties in the gel-drug interaction were evaluated. The intermolecular interaction was observed by FT-IR spectra. The 4VP contained C–N aromatic bond appeared at 1281 cm^−1^ while ibuprofen has C=O from carboxylic group at 1711 cm^−1^ (Supporting Information, Figure S3). The peak of Ibuprofen (C=O) was shifted from 1711 cm^−1^ to 1709 cm^−1^ due to interaction with pyridine and C–N peak shifted 1281 cm^−1^ to 1288 cm^−1^. These could be confirmed the interaction between 4VP and ibuprofen. However, the introducing moiety of interaction unit would be very small and it was difficult to find the peak shift from the gel formulation. Likewise, the FT-IR of mixture VBAC and ibuprofen in d-limonene shown the carbonyl significant shifted from 1711 cm^−1^ to 1726 cm^−1^ (Supporting Information, Figure S4). Additional, intensity of N–H broad peak from vinylbenzyl trimethylammonium chloride at 3381 cm^−1^ decreased after mix with drug. The results are explained by the interaction between cationic ammonium salt. 

### 3.3. Effect of Limonene as Chemical Enhancer and Density of the Network Structure on Permeability 

**Figure 4:**
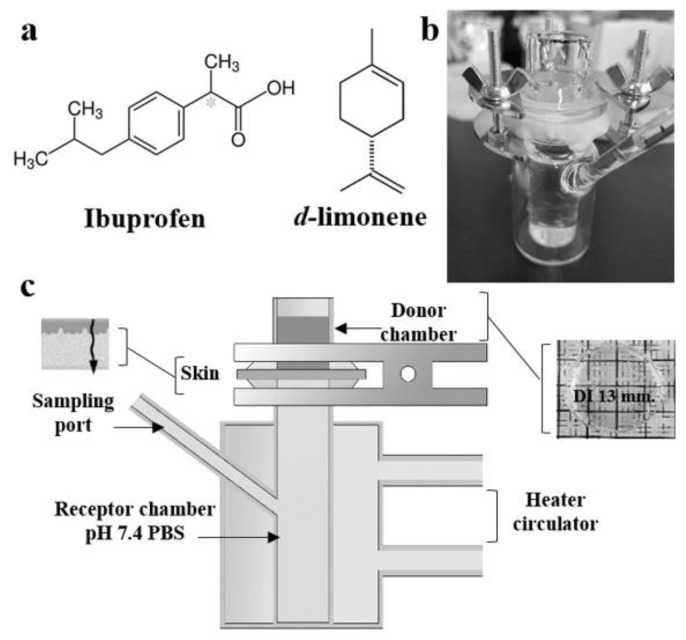
(**a**) Chemical structure of Ibuprofen as model drug and d-limonene, photograph of in vitro permeation experiment by using Franz diffusion cell (**b**) and illustration of permeation study (**c**).

In order to investigate the drug permeation as a controlled release by the cross-linked PS gel swollen in limonene, ibuprofen was selected as a model drug (Figure 4). The drug was released through the rat skin into the receiver solution of pH 7.4 PBS at 37 °C using a Franz diffusion cell as shown in Figure 4c.

**Figure 5:**
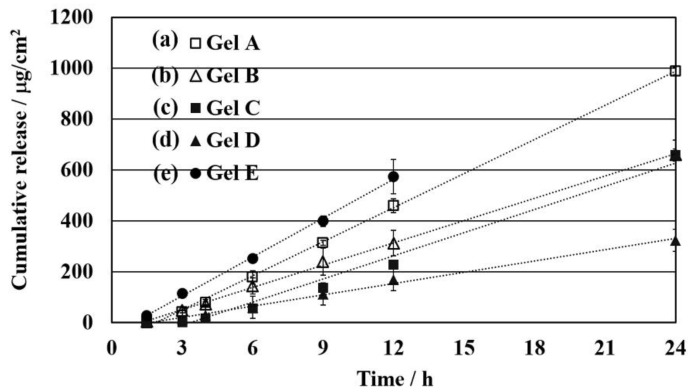
In vitro cumulative release profile of ibuprofen from PS gel; Gel A (**a**), Gel B (**b**), Gel C (**c**), Gel D (**d**) and Gel E (**e**) (*n* = 3).

All PS gels swollen in limonene formulations show high potential releasing properties through the skin because limonene can enhance the permeation of the drug through the epidermis by increasing the activity of drug with SC or decrease the tortuous pathway in SC or both [14]. Nevertheless, the lack of solvent in PS-co-DVB would not be able to permeate drug in transdermal system. These exhibit the advantage of chemical organogel swollen in d-limonene could perform prolong release with simple preparation of cross-linked polystyrene. Permeability of drug from the PS gels swollen in limonene (Table 1, entry 1) was compared to the gel swollen in toluene (Table 1, entry 5) with the similar crosslinking condition (Figure 5a,e).

Toluene can be also defined as chemical enhancer for skin penetration [32]. However, it is extremely toxic and irritating to the skin, showing the merit of the present gels in limonene with the similar enhancer effect (Table 1, entry 1). The PS gels swollen in limonene A-D could prolong controlled release depending on the gel preparation conditions (Table 1, entries 1–4). Additionally, limonene has a low skin irritancy and it allows a reversible change in the skin structure when administered with a pretreatment method [18]. The permeability coefficient (*KD*/*L*) indicates the effect of the enhancer on the diffusion coefficient in SC, whereas the lag time decreases as the diffusion path length decreases. They are determined from Figure 5 and listed in Table 2. During the lag time period, SC would be conditioned for higher permeability and permeation reaches steady state after the lag time. Therefore, the decrease in lag time could also be due to fast SC conditioning times [8]. Gel A showed higher flux and the permeation coefficient as 53.1 ± 0.03 × 10^−4^ cm/h (Table 2, entry 1) than that of gel B as 35.8 ± 0.30 × 10^−4^ cm/h (Table 2, entry 2), influenced of the difference of the crosslinking ratios with 5% (Table 1, entry 1) and 10% (Table 1, entry 2), respectively. The same tendency was observed between gel C as 36.4 ± 0.12 × 10^−4^ cm/h (Table 2, entry 3) and gel D as 17.9 ± 0.20 × 10^−4^ cm/h (Table 2, entry 4) which were prepared both under 8 M with 5% (Table 1, entry 3) and 10% crosslinker (Table 1, entry 4), respectively. The concentration of gel preparation condition was also recognized compared with gel A as 53.1 ± 0.03 × 10^−4^ cm/h (Table 2, entry 1) and gel C as 36.4 ± 0.12 × 10^−4^ cm/h (Table 2, entry 3), which prepared with the same 5% crosslinking ratio (Table 1, entries 1 and 3). It is probably due to the cross-linked network structure of PS gel on activity through gel network. The mobility of drug solute in the skin is represented as D/L^2^ diffusion parameter. It raised when increase the cross-linked degree, gel A as 0.10 ± 1.03 h^−1^ (Table 1, entry 1) and gel B 0.13 ± 0.58 h^−1^ (Table 1, entry 2) for 4 M and gel C as 0.05 ± 1.06 h^−1^ (Table 1, entry 3) and gel D 0.14 ± 0.46 h^−1^ (Table 1, entry 4) for 8 M.

In order to clarify the relationship of permeability and gel network structure, the cross-linker ratio was plotted against to the steady flux of ibuprofen permeation through the skin (*J*_ss_) and elastic modulus (G’) as shown in Figure S5. The steady flux decreased when the cross-linking degree and moduli increased for both concentration (4 and 8 M).

### 3.4. Effect of the Cationic Moiety on the Controlled Release of Ibuprofen for Permeation through the Skin

**Figure 6:**
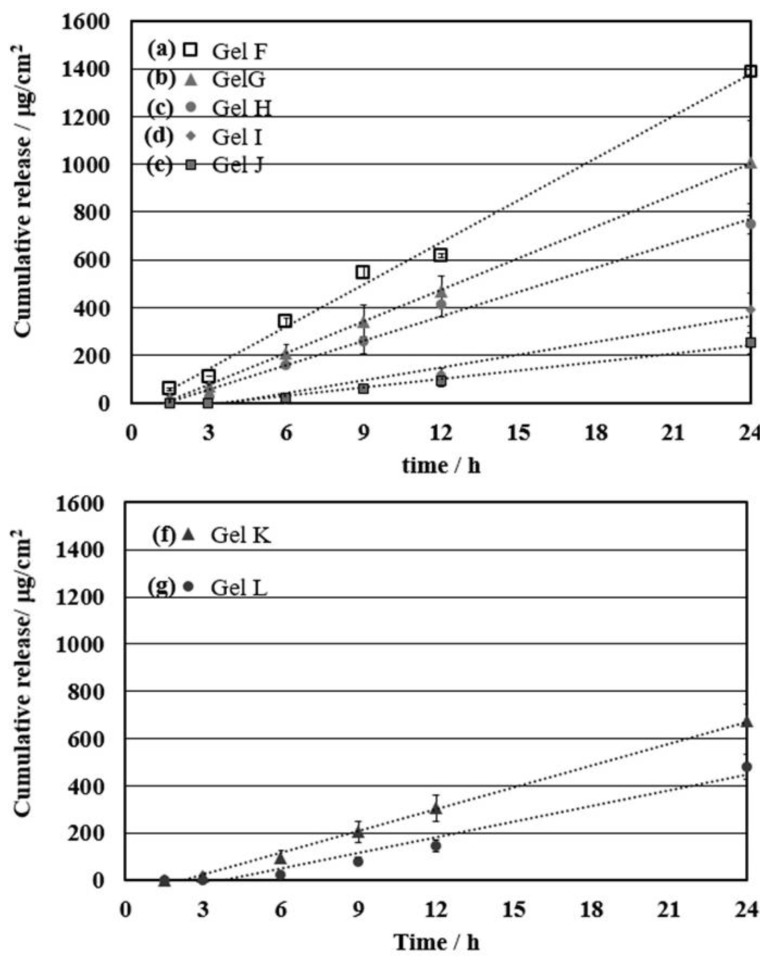
In vitro cumulative release profile of ibuprofen from PS-4VP gel by various feeding ratio of 4VP from 2–5 mol %; Gel F (**a**), Gel G (**b**), Gel H (**c**), Gel I (**d**), Gel J (**e**) and PS-VBAC gel by various feeding ratio of VBAC 0.5 and 1 mol %; Gel K (**f**) and Gel L (**g**) (*n* = 3).

Since the sustainable controlled release is one of the concerning factors in drug delivery systems, it is important to fabricate controllable materials for TDD by organogels. The 4-vinylpyridine and (vinylbenzyl) trimethylammonium chloride were incorporated in order to control the permeation of drug through the skin by interacting with drug molecules. Figure 6 showed the plot of the released amount of the drugs against time and then the permeability (*KD*/*L*), diffusion parameter (*D*/*L*^2^) and lag time were calculated as shown in Table 3. Upon the gradual increased introduction of 4VP with 2 to 5%, the permeability of PS-4VP gels was reduced significantly from 67.1 ± 0.10 to 14.1 ± 0.25 cm/h (Table 3, entries 1–5). Moreover, the lag time tended to increase together with the cationic interaction moiety while diffusion parameter (*D*/*L*^2^) trend to decrease from 0.32 ± 1.14 h^−1^ to 0.04 ± 0.93 (Table 3, entries 1–4). The increase in lagging time and the decrease in permeability and diffusion was attributed to the higher interaction of the cationic moiety with the drug molecules resulting in a controllable drug release.

To emphasize the effect of interaction between drug and gel network structure, a stronger cationic moiety as ammonium salt was introduced as interaction unit in polymer chain in PS gel producing PS-VBAC gel. These interaction force of cationic moiety and ibuprofen (drug) was investigated by FT-IR in Figure S3,4. The VBAC was incorporated only 0.5 and 1 mol % due to poor solubility in d-limonene. However, it is not strong effect to lagging time and diffusion parameter, it was shown insignificantly difference of D/L^2^; 0.07 ± 0.40 h^−1^ and 0.06 ± 0.18 h^−1^, respectively (Table 3, entries 6 and 7). Resulting in the small amount of cationic moiety is not effect to the mechanism of permeation through the stratum corneum. The effect of cationic moiety was still stronger than in PS-4VP gel. Gel K and L was shown the permeability with 37.4 ± 0.38 and 26.9 ± 0.29 cm/h, respectively (Table 3, entries 6 and 7), while Gel F, G and H showed 67.1 ± 0.10, 53.7 ± 0.88 and 41.8 ± 0.21 cm/h (Table 3, entries 1–3). It was indicated that the PS-VBAC gels included smaller amount of cationic moiety (0.5–1 mol %-VBAC) could possess higher efficiency on their prolonged release of ibuprofen than that of PS-4VP gels. 

## 4. Conclusions

In conclusion, the limonene oil gels were successfully fabricated from cross-linked PS and its derivatives, which included 4-vinylpyridne and (vinylbenzyl) trimethylammonium chloride. They were prepared for TDS by using the advantages of d-limonene as an effective chemical permeation enhancer and a low-toxic organic solvent. The stable chemical network of the PS gels swollen in limonene illustrated the highest elastic modulus at 98 kPa. Moreover, the efficiency of the permeability of ibuprofen was successfully enhanced by d-limonene and the controllable of the network density indicated by relative permeability coefficient from 53.1 ± 0.03 to 17.9 ± 0.20 × 10 cm/h. Cationic moieties were introduced to control the drug releasing behavior of the gel with the slowest steady flux at 14.1 ± 0.25 cm/h of 5 mol %-4VP of PS-4VP gel because of drug-polymer chain interaction. However, upon increasing the cationic moiety repulsive effects appeared. Overall, we achieved a steady controlled release of ibuprofen from modified PS gels swollen in limonene.

## Figures and Tables

**Table 1:** Gel preparation and elastic modulus (G’).

Entry	Sample	Gel Type	Ratio of PS_n_-DVB_m_-SD_p_ (n: m:p) ^a^	Comonomer	Comonomer (mol %)	Conc. (M)	G’ (Pa)
1	Gel A	PS gel	95:5:0	-	-	4	680
2	Gel B		90:10:0	-	-	4	6800
3	Gel C		95:5:0	-	-	8	44,200
4	Gel D		90:10:0	-	-	8	98,600
5	Gel E ^b^		95:5:0	-	-	4	1600
6	Gel F	PS-4VP gel	95:5:2	4VP	2	4	2600
7	Gel G		95:5:2.5	4VP	2.5	4	10,300
8	Gel H		95:5:3	4VP	3	4	11,700
9	Gel I		95:5:4	4VP	4	4	21,300
10	Gel J		95:5:5	4VP	5	4	14,000
11	Gel K	PS-VBAC gel	95:5:0.5	VBAC	0.5	4	5000
12	Gel L		95:5:1	VBAC	1	4	1400

^a^ the n, m and p are referred to the feeding ratio of St, DVB and SD (4VP and VBAC), respectively from PS_n_-DVB_m_-SD_p_ formulation.

^b^ Gel E was polymerized in toluene, while all the other gels were polymerized in limonene.

**Table 2:** The skin permeation parameters of PS gel (gel A–D) and toluene gel (gel E).

Entry	Gel	Flux, *J*_ss_ (μg/cm^2^⋅h)	Lag Time (h)	*D*/*L*^2^ (h^−1^)	*KD*/*L* (*10^−4^ cm/h)
1	Gel A	43.7 ± 0.3	1.72 ± 0.16	0.10 ± 1.03	53.1 ± 0.03
2	Gel B	29.4 ± 2.5	1.31 ± 0.29	0.13 ± 0.58	35.8 ± 0.30
3	Gel C	29.9 ± 1.0	3.15 ± 0.16	0.05 ± 1.06	36.4 ± 0.12
4	Gel D	14.7 ± 1.6	1.18 ± 0.36	0.14 ± 0.46	17.9 ± 0.20
5	Gel E	50.9 ± 5.5	0.91 ± 0.16	0.18 ± 1.06	61.9 ± 0.67

**Table 3:** The skin permeation parameters of ibuprofen from PS-4VP gel (gel F–J) and PS-VBAC (gel K and L).

Entry	Gel	Mol % of Cationic Moiety	Flux, *J*_ss_ (μg/cm^2^⋅h)	Lag Time (h)	*D*/*L*^2^ (h^-1^)	*KD*/*L* (*10^−4^ cm/h)
1	Gel F	2	55.2 ± 0.80	0.51 ± 0.15	0.32 ± 1.14	67.1 ± 0.10
2	Gel G	2.5	44.2 ± 7.22	1.22 ± 0.37	0.14 ± 0.45	53.7 ± 0.88
3	Gel H	3	34.4 ± 1.71	1.67 ± 0.48	0.10 ± 0.35	41.8 ± 0.21
4	Gel I	4	18.1 ± 3.13	3.77 ± 0.18	0.04 ± 0.93	22.0 ± 0.38
5	Gel J	5	11.6 ± 2.03	3.27 ± 0.08	0.05 ± 2.00	14.1 ± 0.25
6	Gel K	0.5	30.8 ± 3.14	2.22 ± 0.42	0.07 ± 0.40	37.4 ± 0.38
7	Gel L	1	22.1 ± 2.42	3.02 ± 0.90	0.06 ± 0.18	26.9 ± 0.29

## Supplementary Materials

The following are available online at http://www.mdpi.com/2073-4360/10/11/1200/s1, Figure S1: HPLC chromatogram of ibuprofen in PBS solution with 6.5 μg/mL (**A**), 13 μg/mL (**B**), 26 μg/mL (**C**), 46 μg/mL (**D**) and standard calibration curve of peak area and concentration (**E**), Figure S2: Schematic illustration of the interaction with drugs and scheme of polymer network with and without cationic moieties at the interface of aqueous media, Figure S3: (**A**) FT-IR spectrum of 4-vinylpyridine (**a**), *d-*limonene (**b**), Ibuprofen (**c**), and 4-vinylpyridine: ibuprofen, 1:1 in limonene (**d**) and (**B**) the expected intermolecular interaction between 4-vinylpyridine and ibuprofen, Figure S4: (**A**) FT-IR spectrum of (vinylbenzyl) trimethylammonium chloride; VBAC (**a**), *d-*limonene (**b**), Ibuprofen (c), and VBAC: ibuprofen, 1:1 in limonene (**d**) and (**B**) the expected intermolecular interaction between VBAC and ibuprofen. Figure S5: The correlation of cross-linker ratio between 5 and 10 mol % against to elastic moduli (G’) and steady flux permeation of PS gel (Gel A–D) with 4 and 8 M limonene concentration and Table S1: Storage modulus and permeation parameter of PS gel.
