# Supplementary material for: Cationic Moieties in Polystyrene Gels Swollen with d-Limonene Improved Transdermal Delivery System
Source: Polymers (Basel). 2018 Oct 27;10(11):1200. doi: 10.3390/polym10111200 (PMC6290618; doi:10.3390/polym10111200)
Supplement: Supplementary file 1 [file polymers-10-01200-s001.pdf]

Supporting information

Cationic Moieties in Polystyrene Organogels Swollen with D-limonene Improved Transdermal Delivery System.

Authors: ***Preeyarad Charoensumran[1], Hiroharu Ajiro[1,2]***

Affiliations:

[1]*Graduate School of Materials Science, Nara Institute of Science and Technology, 8916-5 Takayama-cho, Ikoma, Nara 630-0192, Japan.*

[2]*Institute for Research Inititatives, Nara Institute of Science and Technology, 8916-5, Takayama-cho, Ikoma, Nara 630-0192, Japan*

In this supporting information, there are 4 pages content, 5 figures and 1 table.

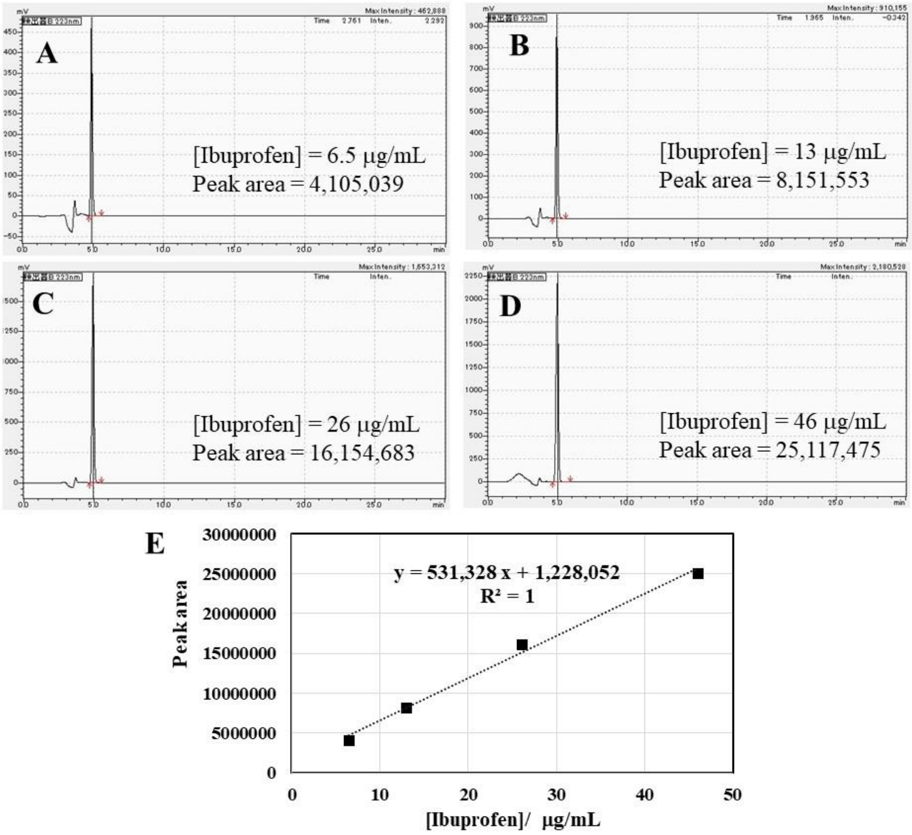

Fig. S1. HPLC chromatogram of ibuprofen in PBS solution with 6.5 μg/mL (A), 13 μg/mL (B), 26 μg/mL (C), 46 μg/mL (D) and standard calibration curve of peak area and concentration (E).

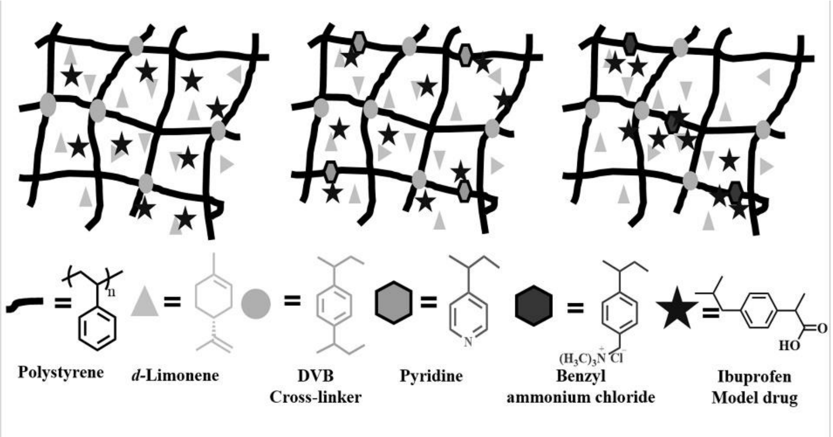

Fig. S2. Schematic illustration of the interaction with drugs and scheme of polymer network with and without cationic moieties at the interface of aqueous media.

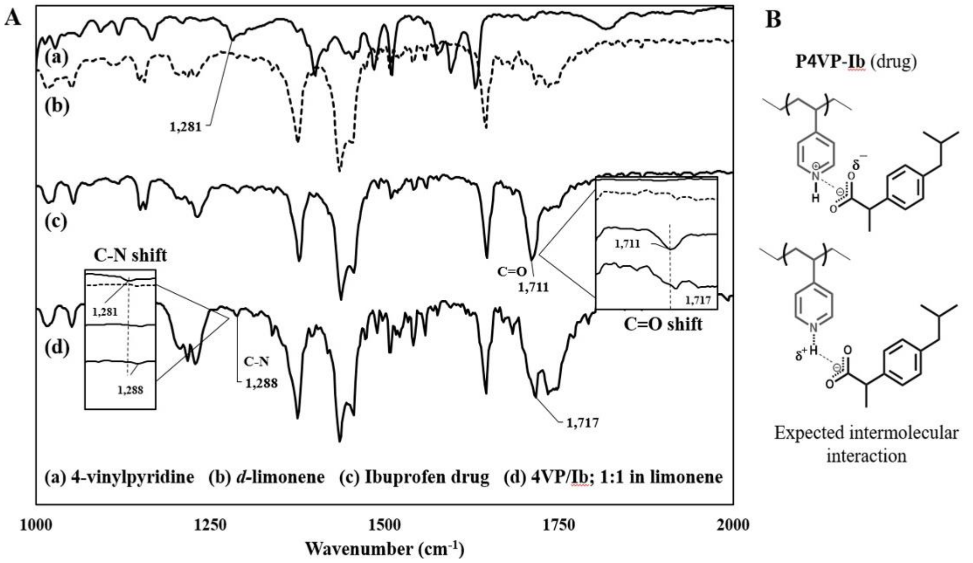

Fig. S3. (A) FT-IR spectrum of 4-vinylpyridine (a), *d*-limonene (b), Ibuprofen (c), and 4-vinylpyridine: ibuprofen, 1:1 in limonene (d) and (B) the expected intermolecular interaction between 4-vinylpyridine and ibuprofen.

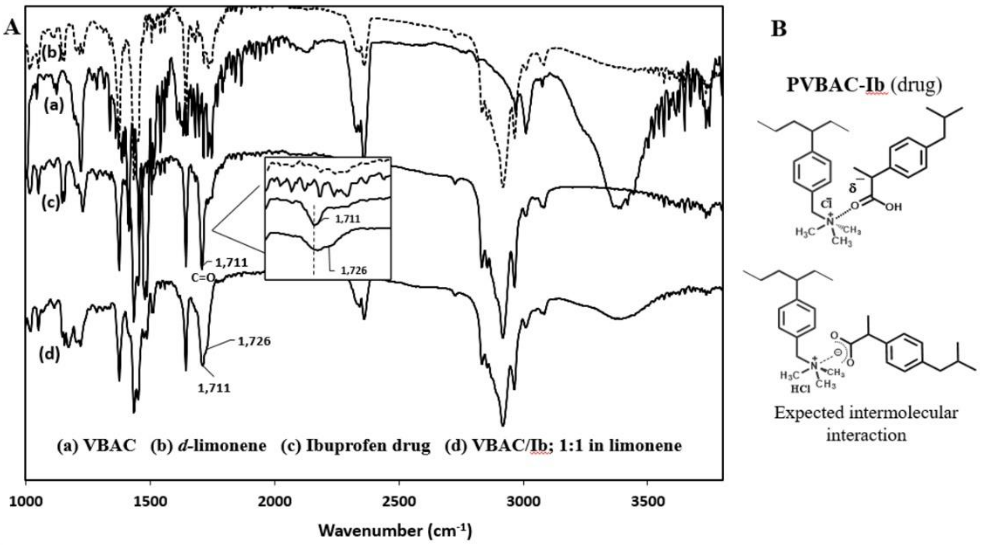

Fig. S4. (A) FT-IR spectrum of (vinylbenzyl) trimethylammonium chloride; VBAC (a), *d*-limonene (b), Ibuprofen (c), and VBAC: ibuprofen, 1:1 in limonene (d) and (B) the expected intermolecular interaction between VBAC and ibuprofen.

The FT-IR spectrum of cationic monomer and drug in *d*-limonene were evaluated to confirm the interaction between 4-vinylpridine and ibuprofen (drug) in Fig. S3 and (vinylbenzyl) trimethylammonium chloride with drug in Fig. S4. The interaction could

perform by electrostatic interaction and H-bond. The 4-vinylpyridine contained C-N aromatic bond appeared at 1,281 cm^{-1} while ibuprofen has C=O from carboxylic group at 1,711 cm^{-1} in Fig. S1A. The carbonyl peak shifted from 1,711 cm^{-1} to 1,717 cm^{-1} (in set Fig. S3A) and C-N peak shifted 1,281 cm^{-1} to 1,288 cm^{-1} (in set Fig. S3A). These could be confirm the interaction between 4-vinylpyridine and ibuprofen as expected in Fig. S2B. However, the introducing moiety of interaction unit are trifling. It is cannot found the peak shift from the gel formulation. Likewise, the FT-IR of mixture VBAC and ibuprofen in *d*-limonene (Fig. S4) shown the carbonyl significant shifted from 1,711 cm^{-1} to 1,726 cm^{-1} (in set Fig. S4A). The resulting are explained by the interaction between cationic ammonium salt and ibuprofen as expected in Fig. S4B.

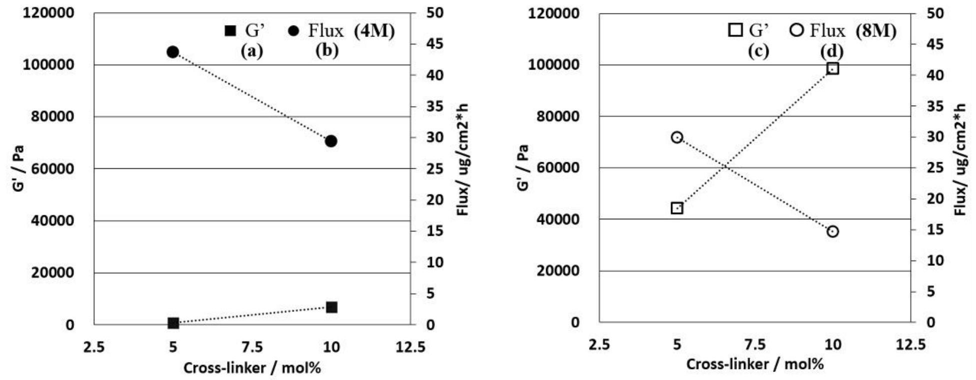

Fig. S5. The correlation of cross-linker ratio between 5 and 10 mol% against to elastic moduli (G') and steady flux permeation of PS gel (Gel A-D) with 4 and 8 M limonene concentration.

Table S1. Storage modulus and permeation parameter of PS gel**.**

Entry	Gel	Cross linker (mol%)	Conc.	Elactic modulus (G')	Flux, J_{ss} (μg/cm^2·h)
1	A	5	4	680	43.7±0.25
2	B	10	4	6,700	29.4±2.47
3	C	5	8	44,200	29.9±1.02
4	D	10	8	98,000	14.7±1.61
